# Towards Clinical Applications of Anti-endotoxin Antibodies; A Re-appraisal of the Disconnect

**DOI:** 10.3390/toxins5122589

**Published:** 2013-12-18

**Authors:** James C. Hurley

**Affiliations:** 1 Rural Health Academic Center, Melbourne Medical School, University of Melbourne, Parkville 3010, Australia; 2 Division of Internal Medicine, Ballarat Health Services, Ballarat 3350, Australia; E-Mail: jamesh@bhs.org.au; Tel.: +61-3-5320-4322; Fax: +61-3-53-206500; 3 Infection Control Committees, St John of God Hospital and Ballarat Health Services, Ballarat 3353, Australia

**Keywords:** endotoxin, endotoxemia, anti-endotoxin antibodies, sepsis, polymyxin, Gram-negative bacteria

## Abstract

Endotoxin is a potent mediator of a broad range of patho-physiological effects in humans. It is present in all Gram negative (GN) bacteria. It would be expected that anti-endotoxin therapies, whether antibody based or not, would have an important adjuvant therapeutic role along with antibiotics and other supportive therapies for GN infections. Indeed there is an extensive literature relating to both pre-clinical and clinical studies of anti-endotoxin antibodies. However, the extent of disconnect between the generally successful pre-clinical studies *versus* the failures of the numerous large clinical trials of antibody based and other anti-endotoxin therapies is under-appreciated and unexplained. Seeking a reconciliation of this disconnect is not an abstract academic question as clinical trials of interventions to reduce levels of endotoxemia levels are ongoing. The aim of this review is to examine new insights into the complex relationship between endotoxemia and sepsis in an attempt to bridge this disconnect. Several new factors to consider in this reappraisal include the frequency and types of GN bacteremia and the underlying mortality risk in the various study populations. For a range of reasons, endotoxemia can no longer be considered as a single entity. There are old clinical trials which warrant a re-appraisal in light of these recent advances in the understanding of the structure-function relationship of endotoxin. Fundamentally however, the disconnect not only remains, it has enlarged.

## 1. Introduction and Overview

**Figure 1:**
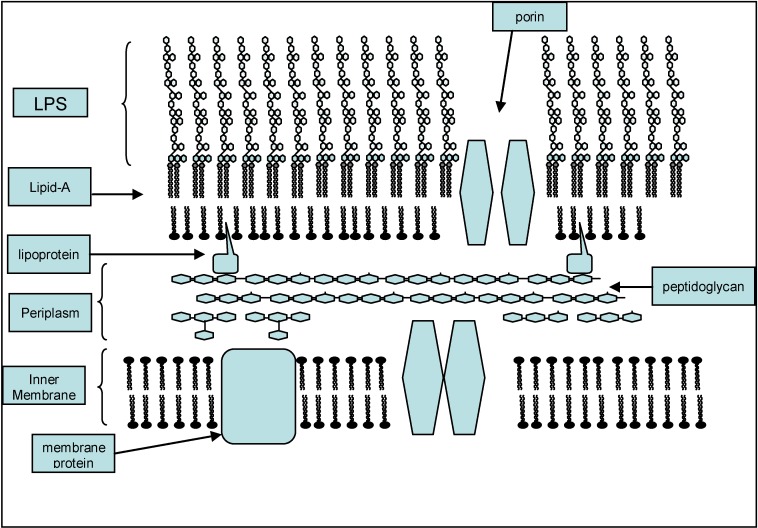
The location of the lipopolysaccharide (endotoxin) molecule in the cell wall of Gram negative bacteria.

Endotoxin is the biological activity that is potential within the Lipopolysaccharide (LPS) macromolecule which is a major outer membrane component of Gram-negative (GN) bacteria (Figure 1) [1,2,3]. Each *E. coli* bacterial cell has approximately 10^6^ LPS molecules [3]. The biological activities of endotoxin in humans and other species are potent and broad ranging. These activities are mediated mostly by the lipid-A residue within the molecule. For these reasons endotoxin has long been identified not only as a potential marker of GN infection [4,5] but also as a mediator and hence a potential target for specific anti-endotoxin therapies [6,7,8,9,10].

Surprisingly however, these potentials have not been realized. The conflicts among over 100 studies of endotoxin as a potential marker of GN infection are reviewed elsewhere [4]. The focus of this review are the disconnects within the literature bearing on the relationship between endotoxemia and sepsis on the one hand and in assessing the potential of anti-endotoxemia therapies including antibody therapy designed to neutralize the biological activity of endotoxin on the other. There is an extensive literature relating to O-polysaccharide specific antibodies generated by vaccination which mediate protection through an antibacterial effect which is not considered here.

## 2. Structure Activity

**Figure 2:**
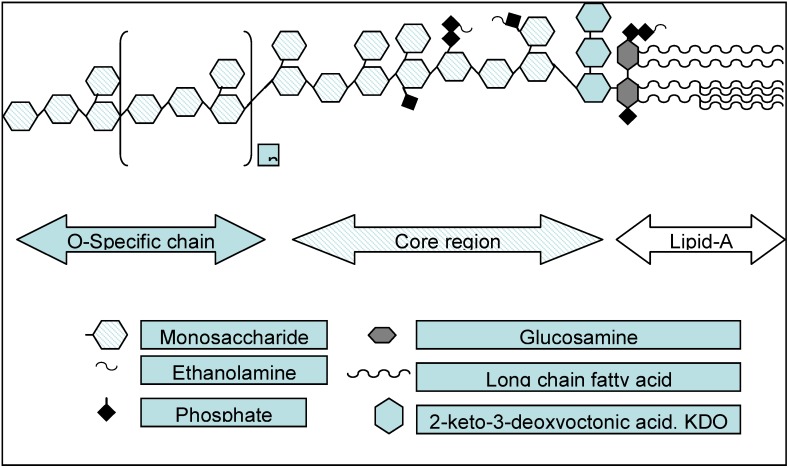
The components of the lipopolysaccharide (endotoxin) molecule.

The term “endotoxin” has been attributed to Richard Pfeiffer. In the 1890’s he made the distinction between the toxic properties that were endogenous within the GN bacterial cell, which he termed “endotoxin” *versus* those released outside the cell, which were termed “exotoxin” [11]. The exact identity of the endotoxin molecule was unknown for several decades and the structure-activity relationship in the mediation of the biological activities of endotoxin has only recently become clear. In the mid 1980’s the lipid-A moiety of the lipopolysaccharide molecule of *Escherichia coli* was totally chemically synthesized in a form available for studies of the structure activity relationship of this molecule [12]. With these studies, it became apparent that the biological activities of endotoxin could be attributed to the lipid-A component of the lipopolysaccharide (endotoxin) molecule (Figure 2). The study of lipid-A partial structures have further clarified this structure-activity relationship [13]. Other microbiological and biochemical studies have identified the mechanisms regulating the synthesis of lipid-A within Gram-negative bacteria and specific variations in the structure of lipid-A which may have relevance to the pathogenesis of GN infection [14,15].

In studying endotoxin and the effects of anti-endotoxin interventions, several pharmacological properties of endotoxin that are unusual for a toxin should be noted. Firstly, there are numerous effects induced by administration of endotoxin to experimental animals and humans. Which of these effects is the most relevant correlate of survival in sepsis is sometimes not clear [16]. Recent review articles list over 20 humoral, cellular, immunological and metabolic effects. Second, unlike most other toxins, the biological effects of endotoxin are not a uniform gravimetric property of the molecule. The quantity of endotoxin is usually interpreted as the amount of biological activity in comparison to a reference endotoxin preparation assayed in parallel. For this reason the concentration of endotoxemia is confusingly and variably reported in weight units (picograms) and sometimes in units of endotoxin activity (Endotoxin Units; EU/mL). The equivalence is dependent on the choice of reference endotoxin but is often approximated as 1 EU/mL ≈ 100 pg/mL [4].

Endotoxin has several other interesting properties. It has long been recognized that human plasma accentuates some of the effects of endotoxin [17,18,19,20]. Moreover, the toxicity of endotoxin is indirectly mediated and endotoxin itself is not cytotoxic, a phenomenon which is best exemplified in the C3H/HeJ mouse strain [21,22]. This inbred mouse strain is resistant to the effects of endotoxin and this non-responsiveness is a genetically determined trait. Moreover, the susceptibility to endotoxin can be restored by the transfer of macrophage cells in bone marrow transplants from histo-compatible C3H/HeN strain mice which have normal endotoxin responsiveness. This observation implicated the mediation of the effects of endotoxin by a genetically encoded host receptor. This observation ultimately enabled the identification of the gene for the endotoxin recognition (*lps*), and in turn, the LPS receptor mechanisms.

Endotoxin has potent biological activities in humans [23,24,25]. In humans, biological responses are apparent at doses as small as 4 ng/kg [25] whereas in other species doses of 1 mcg/kg (rabbits [26], sheep [27]) or as high as 2 mg/kg (rats [28], pigs [29], and non-human primates [30,31,32,33]) are required for response. The difference in endotoxin activity between species is a confounding factor in preclinical studies in animals towards defining the role of endotoxin as a mediator in the pathogenesis of GN infections and in the development of anti-endotoxin antibodies. The curious extreme sensitivity of humans to the effects of endotoxin is problematic for the development of anti-endotoxin therapies in that replication in experimental animals requires either large doses of endotoxin or artificial interventions to sensitize the experimental animals to the effects of endotoxin. One such intervention is the administration of galactosamine to mice to achieve levels of sensitivity at doses of endotoxin that are comparable to that seen in humans [28].

**Tolerance**. A further biological property of endotoxin that is most unusual for a toxin is tolerance [34,35,36,37,38,39]. Doses of endotoxin in experimental animals that would otherwise be lethal can be tolerated after pre-treatments by sub-lethal doses [34]. In the 1920’s, bacterial preparations containing endotoxin were in therapeutic use to induce a fever as a therapy for cancers and syphilis in humans [37]. Humans, as with other species, will acquire tolerance on repeated dosing. Endotoxin doses as much as 200-fold higher may be required to achieve a fever response equivalent to that achieved with the initial dose. There was a series of fascinating experiments undertaken initially in rabbits and later in humans [38,39,40] to investigate the relevance of tolerance toward GN infections.

Studies were undertaken in the 1960’s using prisoner volunteers who were rendered tolerant to endotoxin by repeated doses of endotoxin and who were subsequently deliberately infected with *Salmonella typhi* (*i.e.*, typhoid) or Pasteurella tularensis (*i.e.*, tularemia). These studies demonstrated that tolerance to endotoxin could be induced by administration of Salmonella endotoxin even during the course of an experimental typhoid illness [38]. Moreover, “*Despite unequivocal activation of the endotoxin tolerance mechanisms……, the febrile and toxic course of typhoid fever proceeded unabated*” [38]. There is some evidence that anti-endotoxin antibodies contribute to endotoxin tolerance [40].

There is an historical footnote to these studies with *Pasteurella tularensis*. The role of endotoxin in the pathogenesis of tularaemia has recently been investigated and found to be complex. While the LPS of this organism is considered to be an important virulence factor, surprisingly the immuno-biological activity of this molecule has been found to be weak [41].

**LPS structure**. The lipopolysaccharide molecule from all clinically relevant GN bacteria studied consists of three components against which an antibody could be directed. These are; a polysaccharide chain, a core oligosaccharide and a lipid component, lipid-A (Figure 2) [11,24]. The LPS molecule, and in particularly, the lipid-A component, has been described as an “information rich” molecule, with many possible sites for specific recognition by prokaryotic and eukaryotic proteins. The structure of the polysaccharide chain of the LPS molecule is highly variable even within species of GN bacteria whereas the lipid-A molecule is broadly conserved across GN bacteria of different types (see below) [24]. The core regions binding sites of LPS being located in the cell wall may be less accessible to antibody binding due to their location in the LPS molecule (Figure 1).

The polysaccharide consists of a repeating saccharide unit in a chain configuration which is hydrophilic and antigenic. Being the outer most part of the LPS molecule, this is presumably more readily accessible to antibody binding than are the core regions of the LPS molecule. In Enterobacteriaceae (e.g., *Escherichia coli*), the polysaccharide is variable in length and in composition between bacterial strains, and confers the bacterial strain’s O-antigen specificity. Some bacterial genera have less complete LPS structures. For example, the O-specific antigenic chain is not present in mucosal pathogens such as *Neisseria*, *Bordetella* and *Haemophilus* and the LPS in these genera have only a core oligosaccharide. The core oligosaccharide can be divided into an inner core and outer core region and for the genus Chlamydia even the outer core region is not present and these genera have only an inner core [3,11].

In developing antibodies to lipoplysaccharide, the site of antibody binding may determine the anti-endotoxin activity. Lipid-A contains the molecular components which critically determine the endotoxic activity of LPS [42,43]. The structure of *E. coli* lipid-A consists of a diglucosamine with two phosphates and six acyl (fatty acid) chains (hexaacyl LPS). Two 3-hydroxymyristate (fatty acid) chains are attached directly to each of the two glucosamines with two secondary (“piggyback”) chains also attached. The fatty acids may be laurate (twelve carbon, C12), myristate (C14) with sometimes palmitate (C16) found as the secondary acyl chain, and the primary (glucosamine-linked) acyl chains typically having 12 carbons. This hexaacyl LPS structure is optimal for recognition by the CD14-MD-2-TLR4 receptor. The LPS molecule from Gram-negative bacteria other than *E. coli* may have more or fewer acyl chains, longer acyl chains, branched acyl chains, unsaturated acyl chains, only one phosphate, or other modifications. The degree of recognition of the various hexaacyl and non- hexaacyl LPS structures by the CD14-MD-2-TLR4 receptor determines the mediation of endotoxic biological activity.

**LPS supra-molecular structure activity**. The structure of the lipid-A monomer also determines its molecular shape [44,45,46]. These shapes are variably conical or cylindrical depending on the ratios between the hydrophobic *versus* hydrophilic regions. The most endotoxically active lipid-A monomer structure is conical whereas less active lipid-A is cylindrical. The activity of endotoxin can be increased by sonication [47]. The biological significance of these supra-molecular structures, and the relative importance of LPS aggregates *versus* LPS monomers towards host recognition, remains unclear.

At concentrations above a critical micelle concentration, lipid-A monomers aggregate into supra-molecular structures determined by the molecular shape of the lipid-A. These supra-molecular structures are sometimes large enough to be viewed under electron microscopy [46].

**Lipopolysaccharide-binding proteins**. Lipopolysaccharide-binding protein (LBP) is a serum protein which binds to the lipid A component of bacterial endotoxin and facilitates its delivery to the CD14 antigen on the macrophage, where pro-inflammatory cytokines are released and a cascade of host mediators is initiated. The neutrophil granular protein bactericidal/permeability-increasing protein (BPI) competes with LBP for endotoxin binding and functions as a molecular antagonist of LBP- endotoxin interactions [48,49].

The specific nature of this binding between these serum proteins and LPS has prompted attempts to develop synthetic LPS binding peptides for possible anti-endotoxin based treatments of sepsis. Preclinical studies with these synthetic peptides demonstrate that they can combine excellent selectivity for LPS binding together with suppression of LPS-induced cytokine release *in vitro* and protection from lethal LPS induced septic shock *in vivo* [50,51,52]. Moreover, the mean concentration required to inhibit growth (mean inhibitory concentration, MIC) and the concentration required to achieve anti-LPS activity with these peptides are comparable [51,52]. Interestingly, the molecular interaction between these peptides and LPS which results in this neutralization of biological activity is evident as a bio-physical change in the LPS supra-molecular structure. With peptide binding, the lipid-A part of LPS is converted from its “endotoxic” conformation, being the cubic aggregate structure, to an inactive multi-lamellar structure. Peptides that bind to lipid-A have direct anti-bacterial activity as a consequence of the key structural role of lipid-A within the bacterial membrane structure.

**LPS molecular structure activity**. Whereas remarkably diverse lipid-A structures are found in the LPS molecules produced by different Gram-negative bacteria, the requirements for maximal activation of animal cells are rather restricted [53,54,55,56,57]. The lipid-A structure that has been most extensively studied and the one that is optimally sensed by the CD14-MD-2-TLR4 receptor is that from *E. coli*. This lipid A consists of a bis-phosphorylated di-glucosamine to which are attached six saturated fatty acyl chains with lengths of 12 or 14 (occasionally 16) carbons. LPS molecules that have this structure are commonly referred to as “hexaacyl LPSs” in contra-distinction to other lipid-A structures which have four, five or seven (*i.e.*, non-“hexaacyl LPSs”) fatty acyl chains.

**Figure 3:**
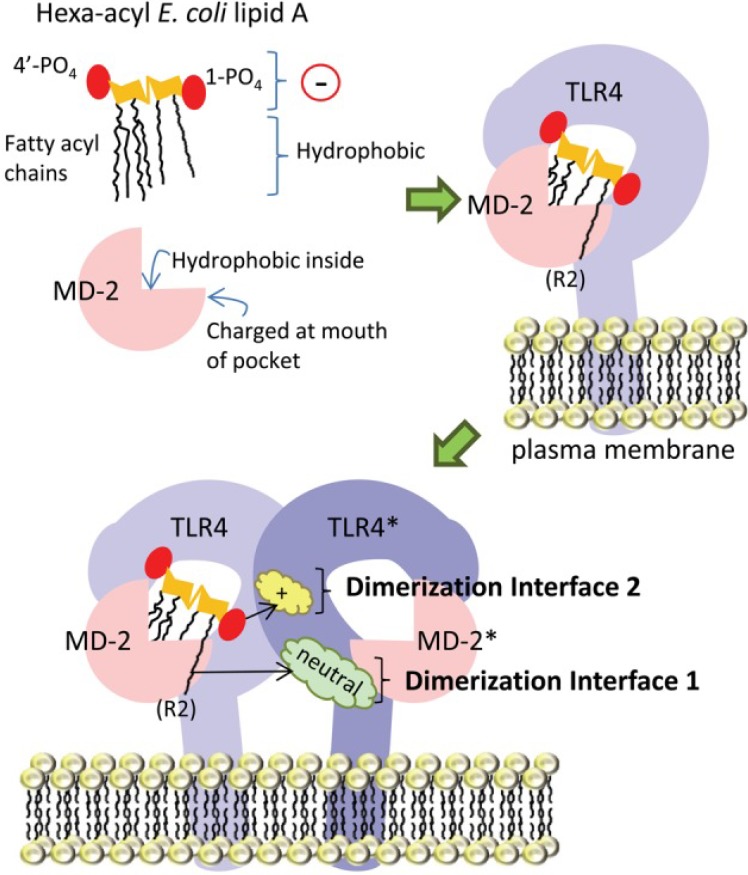
Simplified diagram of TLR4-MD-2 receptor complex dimerization upon ligation of hexa-acyl lipid A. See text for description. Copyright ^©^ 2013 Maeshima and Fernandez. From reference [13], an open-access article distributed under the terms of the Creative Commons Attribution License, which permits use, distribution and reproduction in other forums, provided the original authors and source are credited.

Figure 3 shows the interaction between hexaacyl lipid-A and the CD14-MD-2-TLR4 receptor [13]. The amphipathic lipid A molecule consists of negatively-charged (indicated by “Θ”) phosphate groups and hydrophobic fatty acyl chains. The MD-2 co-receptor protein consists of a “binding pocket” which is lined with hydrophobic amino acid residues and charged amino acids at the mouth of the MD-2 pocket; the former and latter accommodate the fatty acyl chains and the phosphate groups of lipid-A, respectively. The next part of the figure shows lipid-A inside the MD-2 pocket and also in contact with TLR4, with which it forms a 1:1 complex. Upon lipid-A binding, this TLR4-MD-2 complex dimerizes with a second complex. The bound lipid-A engages the TLR4 molecule in the second complex (TLR4*) at two main dimerization interfaces; the first being between the 6th acyl chain of lipid A (which extends out of the pocket) with uncharged amino acids (cloud labeled “neutral”) on TLR4*, the second being between the negatively-charged 1-phosphate (1-PO_4_) on lipid-A with positively-charged amino acid residues (cloud labeled “+”) on TLR4*.

Hexaacyl lipid-A structures are common among Enterobacteriaceae such as *E. coli* whereas non-Enterobacteriaceae, such as *Pseudomonas aeruginosa* may have a non-hexaacyl lipid-A structure. The structural differences in lipid-A among clinical GN bacterial isolates is a relatively recent finding. The possible clinical relevance of these differences between the lipid-A structures of colonizing *versus* environmental GN bacteria remains to be determined.

## 3. Identifying the Target Population

Even were the perfect anti-endotoxin antibody were to be available tomorrow, we would still need to identify the patient population in which it should be used [58,59]. This is generally regarded as the patient population with sepsis. What is sepsis [60]?

Sepsis is a clinical syndrome for which defining objective diagnostic and prognostic tests are lacking and continue to evolve [61,62,63,64]. Forty years ago Lewis Thomas said that “*It is our response that makes the disease*” [63], a comment which succinctly describes sepsis as being the body’s systemic response to suspected or proven infection rather than the infection per se.

In 1991, The American College of Chest Physicians (ACCP) and the Society of Critical Care Medicine (SCCM) convened a panel of 35 experts to derive a standard set of definitions to rapidly identify and triage those patients who might benefit from novel anti-endotoxin antibody therapies that were about to enter clinical trials [65]. The resulting consensus criteria were initially termed the Bone criteria and subsequently evolved into the Systemic Inflammatory Response Syndrome (SIRS) which consists of the presence of at least two of the following;

- Body temperature >38 °C or <36 °C;
- Heart rate > 90 beats per minute;
- Respiratory rate > 20 breaths per minute of hyperventilation evident with a PaCO_2_ < 32 mmHg;
- White blood cell count (WCC) >12,000/mm, <4000/mm^3^ or with >10% immature neutrophils.

The main advantage of SIRS and related criteria are their relative simplicity which led to widespread clinical application. Sepsis, severe sepsis and septic shock are common in the ICU setting with each occurring in as many as 10% of ICU admissions [66,67,68,69,70]. Sepsis and septic shock are associated with substantial attributable mortality. In the CUB-Rea database, the risk of death was 54% in those with *versus* 28% in matched controls without septic shock giving an estimated attributable risk of 26% [66].

However, the limitations in using these defining criteria of sepsis are that;

- They are too sensitive, as non-infection related conditions are included [61];
- The sepsis clinical syndrome is non-specific as to whether the causative organism is Gram-negative bacterial or other [64];
- Being a reflection of the host response, SIRS has an associated morbidity and mortality which is similar whether or not the SIRS is associated with a documented infection [67,68]; and
- As many as a third of episodes are found to occur outside of the ICU setting [68].

The limitations of the current methods for defining and testing for sepsis is best illustrated by a contrast with the field of cardiovascular medicine where in the last ten years, the availability of the serum cardiac troponin test, a rapidly available serum test of cardiac injury, has transformed the management of patients presenting with acute coronary syndromes [71]. A rapid test for sepsis with this sensitivity and specificity is not yet available.

A rapid test for endotoxemia would appear to be a logical method for identifying patients with the potential to benefit from anti-endotoxin therapies, and this approach has been used in some studies of anti-endotoxin antibodies. This is to be further explored with newer endotoxemia assays such as the Spectral Endotoxin Activity Assay^®^ as used in the EUPHRATES trial. However to date, the use of a rapid test for endotoxemia as a method for patient stratification has not proved to be practical due to issues relating to the available assays for endotoxemia [4]. Even within the endotoxemia positive sub-groups of patients within clinical trials, the results of studies of anti-endotoxemia therapies have not been as expected (see below).

Moreover the interpretation of endotoxemia assays whether qualitative or quantitative are not simple [4,72,73]. The levels of endotoxemia encountered in patients with sepsis vary over a range of several log-fold. Among patients with meningococcemia this range is from 10 to 10,000 ng/L [74] and among patients with sepsis this range is from undetectable (<2 pg/mL) to 5 ng/mL [4]. Interestingly it has been reported that endotoxemia is detectable in runners at the conclusion of a marathon at levels comparable to those with sepsis [75].

## 4. Endotoxemia as a Therapeutic Target in Sepsis

In the development of anti-endotoxin antibodies as potential therapeutic agents, the following questions need to be considered in their clinical evaluation;

- What do bacteremia and endotoxemia separately contribute towards the attributable mortality of Gram negative sepsis?
- Is GN bacteremia a single entity?
- Can endotoxemia be considered as a single entity?
- Is the attributable mortality dependent on the underlying mortality risk?
- Is the onset of severe sepsis an indication that the detrimental pathophysiological process has passed the point of no return and hence anti-endotoxemia therapy at this point is futile?
- Is the disconnect bridgeable or is a more fundamental explanation required?

None of the above questions can be satisfactorily resolved in an animal model of sepsis. For example, GN bacteremia is clearly not a single entity despite the fact that experimental models commonly have used a single bacterial species, typical *E. coli*, as the challenge inoculum. Amongst various GN bacteremia types there is a well-defined ranking of mortality risk with the risk being lowest for *E. coli* and highest for GN bacteremias such as *Pseudomonas aeruginosa*. Likewise, endotoxemia should not be regarded as a single entity given the recently described differences in the lipid-A structures among the different GN bacteremia types. Also, the relationship between endotoxemia *versus* GN bacteremia both with respect to their mutual concordance and as predictors of outcome among patients with suspected sepsis is not simple [76,77,78].

Endotoxemia is not detectable for at least 20% and up to 50% of patients with GN bacteremia. Moreover, the relevance of endotoxemia detection to prognosis is dependent on the co-detection of GN bacteremia, the GN bacteremia type and the underlying mortality risk in the study population [76,77]. This has been examined in a series of meta-analyses and L’Abbé plots using data derived from 100 studies of patients with sepsis in various settings. The most recent analyses examined the relative strength of endotoxemia and GN bacteremia as independent predictors of increased mortality risk [77] and the confounding relationship with the GN bacteremia type [78].

**Figure 4:**
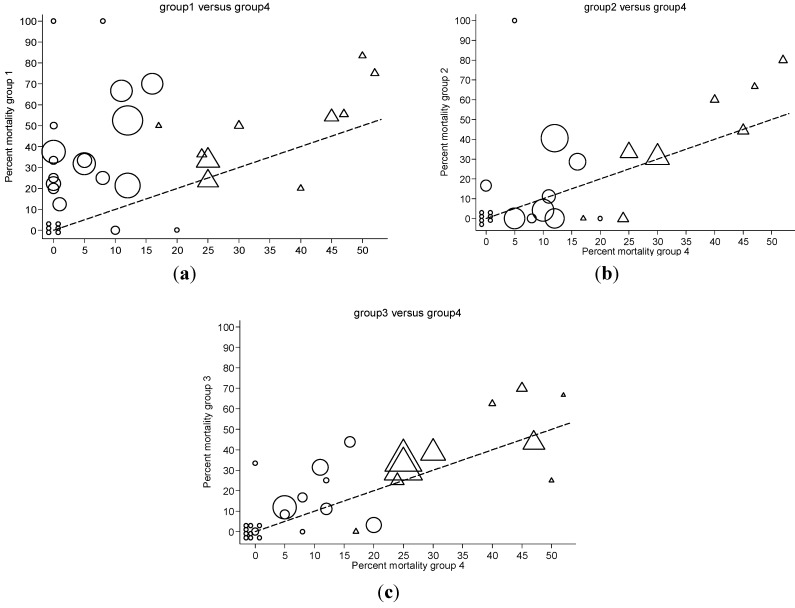
L’Abbé plots of study specific mortality rates from 35 studies. Each figure shows mortality rates for studies undertaken in an ICU (triangles) or non-ICU (circles) setting with symbols proportional to group size with the line of no difference (y = x; dotted line) shown for visual reference purposes. Shown are (**a**) Groups 1 (endotoxemia and GN bacteremia detected) *versus* groups 4 (neither detected); (**b**) Groups 2 (GN bacteremia alone) *versus* groups 4 (neither detected); and (**c**) Groups 3 (endotoxemia alone) *versus* groups 4 (neither detected). GN, gram negative. From [77] ^©^ 2012 Hurley *et al*.; licensee BioMed Central Ltd., an open access article distributed under the terms of the Creative Commons Attribution License.

The most recent of these analyses has attempted to identify the separate contributions toward the impact of endotoxemia *versus* GN bacteremia on the mortality risk among various studies of patients in various settings [77]. The mortality risk was analyzed from 35 studies for which individual patient data was available. This data was stratified into four distinct groups; GN bacteremia and endotoxemia co-detected (patient group 1), GN bacteremia alone (group 2), endotoxemia alone (group 3) each *versus* patients with neither endotoxemia nor GN bacteremia detected (group 4). The difference in mortality risk between group 4 *versus* respectively groups 1, 2 and 3 can be represented in a summary odds ratio (OR) or risk difference (RD). In addition, the heterogeneity in risk across these 35 studies can be visualized in the “scatter” within Figure 4. Also as group 4 is generally the largest group, the mortality risk for this group can be taken as representative of the underlying mortality risk for each study.

The co-detection of endotoxemia with GN bacteremia is most predictive of increased mortality risk (OR 6.9; 4.4–11.0; RD 19%; 11%–27%) *versus* the detection of neither (Figure 4a). However, this was most apparent amongst a sub-group analysis of studies undertaken outside an ICU setting and is a finding which is associated with marked heterogeneity [4,77]. By contrast, the detection of either endotoxemia (Figure 4c) or GN bacteremia (Figure 4b) in isolation *versus* the detection of neither is associated with borderline elevation of risk (OR < 2.0; RD < 10%) and, moreover, is associated in each case with low levels of heterogeneity. This lack of heterogeneity is surprising given the diversity of patient groups, underlying risk, and clinical settings in these 35 studies that were conducted and published over a period exceeding three decades.

The heterogeneity is most apparent in the contrast of mortality risk for patient groups one *versus* four (Figure 4a). In this regard, there is an unequal distribution in GN bacterial isolates among the patients of groups 1 and 2. *E. coli* bacteremia isolates are less common in group 1 *versus* group 2 whereas for *Pseudomonas* bacteremia isolates the converse applies. Moreover, while the underlying patient risk was higher for studies in an ICU setting *versus* studies in other settings (Figure 4), as might be expected, the additional mortality associated the detection of either or both of endotoxemia or GN bacteremia was less apparent in the studies of patients at high *versus* low underlying risk [77].

A further analysis of the individual patient data from studies of patients derived from eight ICU studies and small numbers from an additional 6 non-ICU studies had been undertaken [78]. These patients were identified to have GN bacteremia with or without endotoxemia (*i.e.*, groups one and two). This analysis has unexpectedly revealed that the detection of endotoxemia in association with *E. coli* bacteremia has no prognostic value in any setting [78]. It is notable that this lack of prognostic value with *E. coli* bacteremia is at variance with the experience for GN bacteremia overall. Note that *E. coli* bacteremia is often the most common GN bacteremia category in any clinical series. This finding is also surprising given the extensive use of endotoxin derived from *E. coli* in experimental models. This factor is yet to be explored as a possible basis for the disconnect between the pre-clinical and clinical experience with anti-endotoxin antibodies.

Some have questioned whether the onset of severe sepsis indicates that the detrimental pathophysiological process has passed the point of no return and hence anti-endotoxemia therapy at this point is futile. The answer to this question can only be speculated. However, there are parallels with antibiotic therapy for which there is now clear evidence that early effective antibiotic therapy is critical to optimal activity and survival of patients with septic shock [79] or conversely, late antibiotic therapy could be considered relatively ineffective. Moreover, combination antibiotic therapy may improve survival [80]. The question then becomes what additional benefit from early effective combination antibiotic and anti-endotoxin therapies could be expected? Only an adequately designed clinical trial can answer this question.

There is a more fundamental question. Is it possible to bridge the disconnects between the solid pre-clinical observations of the structure function by which the biological effect of lipid-A are mediated on the one hand *versus* the conflicting evidence for the relationship between endotoxemia and sepsis outcome as has been observed in the clinical setting on the other? In 1993 I questioned the role of endotoxemia in sepsis and proposed an alternative hypothesis [81]. I suggested that the release of endotoxin is a marker of the transition of Gram-negative bacteria to cell wall deficient forms (L-forms) that may persist in an undetected state and in this state would be non-responsive to antibiotic therapy directed against the cell wall intact parental bacterial forms [82,83]. Under this hypothesis, the disconnect could be bridged. It would follow from this hypothesis that anti-endotoxin therapies would have no clinical value in sepsis regardless of any ability to bind to and block the effects of endotoxin activity in pre-clinical studies.

## 5. Anti-endotoxin Therapies and the Disconnect

The term “sepsis disconnect” has been used to describe the failure to translate anti-endotoxin (and other immuno-modulatory) agents from promising pre-clinical evidence to proven clinical therapy [84,85,86,87]. Likewise, a meta-regression analysis asked why “*despite promising pre-clinical testing and the expenditure of several billion dollars, anti-inflammatory agents designed to inhibit specific host mediators failed to show benefit in 22 clinical trials involving over 10,000 patients*” [87]. This meta-regression questioned whether variability in the underlying risk of death between preclinical *versus* clinical studies could be an explanation for the disconnect [87].

However, no simple explanation has emerged to account for this “disconnect” and there are numerous paradoxical discrepant findings between the pre-clinical studies on the one hand and the randomized controlled clinical trials of the same agent on the other [88,89,90,91,92,93]. Hence the term “endotoxin-sepsis disconnect” could be extended to positive results from the early clinical studies with anti-endotoxin therapies which raised expectations for progress in the development of these therapies. Failure to replicate findings with specific anti-endotoxin antibodies in later studies, also represents a paradoxical “disconnect” [91,92]. Anti-inflammatory agents that have been developed to block mediators (e.g., anti-TNF, anti-PAF) whose release is stimulated by endotoxin and hence are “down-stream” are not considered here although these also each exemplify a paradoxical disconnect. Some additional literature is cited and discussed further in [93].

Indeed more broadly, no adjuvant approach to the treatment of sepsis has emerged from the extensive pre-clinical research. This extensive literature dealing with the pre-clinical and clinical evaluations of candidate anti-sepsis agents is complex and difficult to summarize. Some examples of “disconnect” are reviewed here to the extent that they have a bearing on the pre-clinical and clinical evaluation of anti-endotoxin antibodies for adjuvant therapy in Gram-negative sepsis.

### 5.1. Anti-endotoxin Therapies: Pre-clinical Disconnect

Despite 40 years of research with at least six candidate anti-endotoxin antibody therapies each having shown promise in pre-clinical and early clinical trials, no anti-endotoxin antibody approach to the treatment of sepsis has emerged with proven clinical benefit. In relation to anti-endotoxin therapies, the extent to which the mediation of survival benefit in experimental studies with the various anti-endotoxin therapies relate to the binding of endotoxin, the neutralization of endotoxin activity *versus* the clearance of either endotoxemia or even bacteremia are each contentious and unclear [91,94]. For example, a review article which reviewed the evidence for and against one extensively studied hypothesis,—that antibodies to the inner core raised by immunization with enterobacterial deep rough mutants confer broad spectrum protection during Gram-negative bacterial sepsis, cites over 200 references published up to 1997 [95]. The most paradoxical suggestion is that some of the anti-endotoxin monoclonal antibodies and recombinant proteins under study had been contaminated by endotoxin in the production process [96,97]. This endotoxin contamination from the source bacteria could account for the difficulties in replicating the protection in later pre-clinical studies.

A question of particular interest in this contentious area of research is the evidence pertaining to the relationship of survival benefit to reductions in levels of circulating endotoxemia. Unresolved issues that could bridge the pre-clinical *versus* clinical disconnect is the possibility that anti-endotoxin therapies may be more effective in some patient sub-groups if only these could be identified [87]. In this regard the differences in lipid-A structure that have recently been discovered between *E. coli* *versus* other Gram negative bacteria together with the finding that endotoxemia has no impact on mortality when detected in association with *E. coli* in contrast to other types of GN bacteremia remain an important basis for re-appraisal of this old literature [4,78].

Some of the anti-endotoxin therapies including antibodies which have reached clinical studies are summarized in Table 1, Table 2 and Table 3. The agents listed in these tables have anti-endotoxin activity which is thought to be mediated either through binding to lipid-A or through blocking the effects of endotoxin presumably at the level of the receptor. However, even these pre-clinical studies of binding and mechanism of action are controversial and in some cases have been difficult to reproduce [98]. Not shown in these tables is a range of other biological agents designed to inhibit downstream immunological mediators whose release is triggered by endotoxin which have been evaluated in randomized clinical trials of the patient group with sepsis. Recent commentaries [87,99] and a systematic review of 45 reviews of animal studies of therapeutic interventions for sepsis found evidence of over-extrapolation of the preclinical research findings and an under-appreciation of the deficiencies and risk of bias in study methods [100].

**Table 1:** Clinical studies of anti-endotoxin therapies including antibodies: Studies of pre-immunity and prophylaxis.

Author	Year	Reference	Agent	Setting	N	Mort	GNI
McCabe; Zinner	1972, 1976	[101,102]	core Ab ^§^	Hospital	182	↓	ND
Pollack	1983	[103]	core Ab	Hospital	43	↓	ND
Baumgartner	1985	[104]	J5 IVIG	ICU	262	↓*	ND
McCutchan	1983	[105]	J5 IVIG	Oncology	100	↔	↔
Cometta	1992	[106]	J5 IVIG	Surgical	329	↔	↓
Bennett-Guerrero	1997	[107]	core Ab ^§^	Surgical	301	ND	↓

Notes: Mort = mortality; GNI = GN infection incidence; ND = No data; **↓** = decrease; ↓* = decrease noted in a subgroup; **↔** = no significant change; **^§^** = present in acute phase or pre-operative serum.

**Table 2:** Clinical studies of anti-endotoxin therapies including antibodies: Treatment studies restricted to Meningococcal disease.

Author	Year	Reference	Agent	Setting	N	Mort
Meningococcal disease						
J5 study group	1992	[108]	J5 PC	ICU	73	↔
Derkx	1999	[109]	HA-1A	ICU	267	↔
Levin	2000	[110]	rBPI21	ICU	892	↓*

Notes: Mort = mortality; ND = No data; **↓** = decrease; ↓* = decrease noted in a subgroup; **↔** = no significant change.

**Table 3:** Clinical studies of anti-endotoxin therapies including antibodies: treatment studies unrestricted to specific GN infections.

Author	Year	Reference	Agent	Setting	N	Mort	GNI
Polyclonal anti-sera							
Ziegler	1982	[111]	JS PC	Hospital	212	↓	ND
Calandra	1988	[112]	J5 PC	ICU	71	↔	↔
Grundmann	1988	[113]	IVIG	ICU	46	↔	ND
Schedel	1991	[114]	IVIG	Hospital	55 ^§^	↓	ND
Behre	1992	[115]	IVIG	Oncology	21	↓	ND
Behre	1995	[116]	IVIG	Oncology	52	↓	ND
Monoclonal antibodies							
Ziegler	1991	[117]	HA-1A	Hospital	200 ^§^	↓*	ND
Greenman	1991	[118]	E5	Hospital	212	↓*	ND
Fisher	1990	[119]	HA-1A		34	ND	ND
French Reg	1994	[120]	HA-1A	Hospital	600	↑	ND
McCloskey	1994	[121]	HA-1A	Hospital	2199	↔	ND
Angus	2000	[122]	E5	ICU	1090	↔	ND
Daifuku	1992	[123]	MAB-T88		9	ND	ND
Greenberg	1991	[124]	E5				
Greenberg	1992	[125]	E5		39	ND	ND
Albertson	2003	[126]	ECA-Ab	ICU	411	↔	ND
Other endotoxin agents							
Willats	1995	[127]	Taurolidine	ICU	100	↔	↔
Reinhart	2004	[128]	iHSA	ICU	143	↔	ND
Bennett-Guerrero	2007	[129]	E5564	Surgical	152	↔	ND
Tidswell	1910	[130]	E5564	ICU	235	↔	ND
Opal	2013	[131]	E5564	ICU	1961	↔	ND
Dellinger	2009	[132]	PLE	ICU	1379	↔	ND
Heemskerk	2009	[133]	ALP	ICU	36	↔	ND
Pickkers	2012	[134]	ALP	ICU	36	↔	ND

Foot notes: iHSA = human serum albumen; ALP = Alkaline phosphatise; Mort = mortality; GNI = GN infection incidence; ND = No data; **↓** = decrease; ↓* = decrease noted in a subgroup; **↑** = increase; **↔** = no significant change; ^§^ = Two studies are noted here to have a high proportion of non- *E. coli*-Enterobactericeae relative to *E. coli* amongst the GN bacteremias.
Schedel [114], of 55 GN bacteremias 30 were non- *E. coli* Enterobactericeae and 12 were *E. Coli*; Ziegler [117], of 200 GN bacteremias 71 were non- *E. coli* Enterobactericeae and 87 were *E. coli*.

There are complex challenges in developing appropriate animal model systems in which to test anti-endotoxin therapies. Such a model needs to address at least three basic issues; the question of dosages of endotoxin, the importance of route of endotoxin administration in natural and experimental situations, and the importance of the variation of animal species in the type of responses elicited by endotoxin [135]. A range of experimental models exist such as the ceacal perforation peritonitis model [135,136,137]. Which of these several models is optimal toward the evaluation of anti-endotoxin therapies is unclear. A recent finding that the genomic response in a mouse model shows little correlation with that in human sepsis has further reinforced the difficulties in the interpretation of animal model data [138].

Moreover, an optimal animal model needs to be more than simply an “intoxication” model [135,139,140,141]. Any beneficial therapeutic effect would presumably need to be additional to that provided by supportive and other therapies such as antibiotics. The desirable type of study design is one which more closely reflects the sepsis dynamic in the clinical setting but this is difficult to achieve.

Controlled models of septic shock have been developed in dogs and other animals by workers at the National Institutes of Health (NIH) in attempts to disentangle the separate contributions of clearance of endotoxemia *versus* bacteremia toward outcome. These models have generated unexpected and paradoxical observations bearing on the relative importance of clearance of bacteremia *versus* endotoxemia in relation to the effects of experimental therapeutic interventions on outcome [142,143,144,145,146,147,148,149,150,151,152,153,154].

In the dog, implantation of an intra-peritoneal clot infected with various selected Gram-negative and Gram-positive challenge bacteria induces bacteremia together with cardiovascular changes characteristic of septic shock leading to lethality [142]. The outcomes of any therapeutic interventions can then be studied under controlled conditions simulating a clinical trial including randomized treatment assignment and investigator blinding [146]. This model enables the study of the relative effects of intervention with antibiotics, cardiovascular support and anti-endotoxin therapies on hemodynamic changes, survival time, quantitative levels of bacteremia as well as quantitative levels of endotoxemia.

A series of paradoxical observations have emerged from these studies which are of interest to the questions bearing on the relationship of the effect of anti-endotoxin agents to the clearance of bacteremia *versus* endotoxemia and toward improved outcome. Amongst these studies were several that addressed variations in the type of bacterial challenge, the strain type, virulence factors and a range of therapeutic interventions [142,143,144,145] which are reviewed elsewhere [155]. Only those studies that are most relevant to the development of anti-endotoxin therapies including anti-endotoxin antibodies are reviewed here.

A strikingly paradoxical observation arose when the following putative anti-endotoxin interventions were studied; two anti-endotoxin therapies; a monoclonal anti-endotoxin antibody (HA-1A) [146], reconstituted human HDL lipoprotein [152], and plasma exchange [151]. These interventions were each separately studied *versus* control treatments in this model for the effect on survival and the associated levels of bacteremia and endotoxemia following the *E. coli* challenge. The effects of these interventions on the levels of endotoxemia resulted in a lowering [152], or no effect [146], or were not measured [151]. Surprisingly, in each case, the therapies either failed to increase survival or even paradoxically resulted in worsened cardiovascular effects or shortened survival despite similar quantitative levels of bacteremia *versus* control treated animals.

Further studies also generated somewhat unexpected results bearing on bacteremia and endotoxemia in relation to the protective effect of various monoclonal antibodies (MAB) against an *E. coli* challenge in this canine and in other models. Both an isotype matched core region specific MAB and an O-side chain type specific MAB conferred protection additional to that provided by antibiotic and supportive therapy in this canine model [143]. The mechanism of protection differed. For the O-side chain specific MAB protection was mediated through enhanced clearance of the bacteremia and endotoxemia. Whereas the core region specific MAB, while protection was mediated through decreased circulatory collapse, this occurred without any measurable reduction in the bacteremia or endotoxemia compared to control treated dogs.

This NIH group also investigated the protective effect of recombinant granulocyte colony stimulating factor (rG-CSF) *versus* control treatment and the relationship between clearance of bacteremia and endotoxemia and outcome in a canine pneumonia model induced by an *E. coli* intra-tracheal challenge [154]. The enhanced peripheral blood neutrophil counts following prophylactic administration of rG-CSF was associated with reduced levels of endotoxemia and improved survival in this canine model of *E. coli* pneumonia compared to control treatment. The quantitative bacteremia counts were similar in the treatment and control groups.

The summation of these paradoxical observations made over the course of these studies reviewed here together with other studies that have been reviewed elsewhere [4,155] led this group of investigators to speculate as to whether endotoxemia has continued importance once the inflammatory response and the clinical manifestations have been initiated in that possibly… “*tolerance to endotoxemia may develop within hours* [51], *and persistently high levels of endotoxin may not be central to the manifestations of the sepsis syndrome*” [144].

Another research group has also examined a monoclonal anti-endotoxin antibody (HA-1A) as well as an alternate anti-endotoxin intervention, bactericidal/permeability increasing protein (BPI) which is an endogenously produced human endotoxin neutralizing protein, against a lethal *E. coli* at a high (10^11^ cfu/kg inoculum) dose challenge. The plasma endotoxin levels were significantly reduced compared to no treatment after the use of BPI but not with HA-1A. However, whilst both treatments attenuated cytokine release, neither improved survival [156].

### 5.2. Anti-endotoxin Therapies: Clinical Disconnect

The impetus for the development of anti-endotoxin therapies had been supported by a range of clinical observations (Table 1) [157,158]. In the 1970’s, several clinical studies suggested that the presence in acute phase patient serum of high levels of antibodies to core antigens of lipopolysaccharide as expressed in a mutant *E. coli* J5 strain correlated with improved outcome of patients with sepsis [101,102,103]. More recent observational studies have again demonstrated the correlation between the measurable levels of anti-endotoxin core antibodies in pre-operative serum with adverse post-operative events in patients undergoing cardiac surgery [107].

These early observations led to intervention studies using various preparations of anti-sera prepared from volunteer donors specifically immunized using the mutant *E. coli* J5 strain [104,105,106,107]. Anti-sera production was considered to be both an expensive and an impractical undertaking for clinical use outside of investigational studies. Later studies used either immunoglobulin preparations produced commercially from selected blood donations screened on the basis of anti-sera titres or specific monoclonal antibodies.

The results of these intervention studies are contentious as there were a number of different study designs leading to difficulties in interpretation and even reproducibility [157,158]. Among these studies there were differences in anti-endotoxin antibody or agent, differences in study population and differences in study end point. Various preparations of anti-sera or immunoglobulins were used, some containing both IgM and IgG, other preparations only IgG. Some studies used a therapeutic strategy whereas other studies chose to target high risk patient groups as a prophylactic strategy. The end point of interest varied, in some studies being the occurrence of GN bacteremia, this was particularly the case for studies of a prophylactic approach (Table 1). Whereas the end point in other studies was mortality, this was the case more so for studies of a treatment approach (Table 2 and Table 3).

Some of these studies used normal (non-immunized) immunoglobulin preparations as the intervention for control group patients. However, the choice of normal immunoglobulin preparations for control group patients is problematic as it is possible that even these may have anti-endotoxin efficacy in patients with sepsis [115,159,160] even with possible mortality benefit [159], although not seen in all studies [160,161,162,163,164].

A small number of studies listed in Table 1, Table 2 and Table 3 have used the detection of endotoxemia as either a criterion for study entry or as a quantitative outcome of intervention [113,114,115]. In this regard, the studies undertaken in patients with meningococcemia are of particular interest for three reasons. Unlike other Gram negative infections, meningococcemia is clinically distinctive and rapid recognition and early treatment is feasible. Second, endotoxemia occurs more commonly with meningococcaemia than with other Gram negative bacteremias [64]. Finally, there is evidence from Norwegian investigators implicating a relationship between quantitative levels of endotoxemia and outcome among patients with meningococcaemia [74]. However, three studies of anti-endotoxin therapies have failed to find any significant overall mortality benefit in this condition (Table 2). Moreover, one study that examined the effect of endotoxemia detection on outcome using a logistic regression model found no significant interaction effect from the intervention [109] and another study examined levels of endotoxemia but did not report the impact on outcome [110].

There are several possible explanations for the disappointing results in these studies undertaken in patients with meningococcemia. Adequacy of dosing and speed of administration of the intervention have been considered amongst a range of study design aspects. For example, in the 1992 French study [108] a significant increment in the serum level of anti-J5 antibody was not apparent in the intervention group post infusion of anti-J5 antiserum. However, the evidence implicating a relationship between quantitative levels of endotoxemia and outcome amongst patients with meningococcaemia has undergone a reappraisal. Recent studies from the same group of Norwegian investigators has found a close correlation between levels of endotoxemia and bacterial DNA detected using quantitative PCR methods among patients with systemic meningococcal disease [165]. Hence the level of bacteremia is likely to be a confounder in the correlation between the level of endotoxemia and outcome in this condition.

A number of these anti-endotoxin treatment studies have examined the sub-group of patients with detectable endotoxemia for any different treatment response [115,166]. Notable amongst these was a sub-study from the HA-1A monoclonal antibody which concluded that HA-1A reduced mortality amongst the 27 patients with detectable endotoxemia but not amongst 55 patients without detectable endotoxemia [166]. However mostly these have been small sub-groups. For other clinical trials, the results in the endotoxemia positive sub-groups of patients have also not been as expected [131].

One parameter not previously considered in the interpretation of these study results is the type of GN bacteremias seen. Given that the impact of co-detection of endotoxemia with GN bacteremia on patient prognosis is unequal for different types of GN bacteremia, even amongst Enterobacteriaceae [78] it is plausible that a high prevalence of *E. coli* bacteremias among any of the studies in Table 1 and Table 3 could account for the lack of therapeutic success. However, this is speculative and the proportion of *E. coli* bacteremias among the overall total GN bacteremias for most studies is unknown.

In the development and clinical evaluation of anti-endotoxin antibodies, the recent published clinical experience with polymyxin is of interest [167,168,169,170,171,172,173,174,175,176,177,178,179,180]. Polymyxin B is a potential anti-endotoxin intervention as there is strong evidence for endotoxin binding and neutralization in pre-clinical studies. Polymyxins are from a group of cyclic cationic polypeptide antibiotics and these have well characterized lipopolysaccharide binding, which is presumably mediated through binding to lipid-A, and which is associated with inhibition of endotoxin activity as measured *in vitro* and in whole animal studies.

It is notable that the endotoxin antagonist property of polymyxin-B is not simple as it is dependent in part on the bacterial origin of the LPS. Amongst LPS preparations from eight different GN bacteria, Polymyxin was most inhibitory against the LPS of *E. coli* and least inhibitory against the LPS of *N. meningitidis*. An additional paradoxical finding from this study was that while polymyxin at low concentrations was inhibitory to the effects of endotoxin, at concentrations greater than 50 mcg/mL polymyxin acted synergistically with endotoxin in inducing IL-1 secretion [167].

While toxicity limits the clinical use of polymyxin B as an antibiotic, polymyxin B can be bound to a solid phase such as a hemo-perfusion column [169]. This enables hemo-perfusion as a method for the removal of circulating endotoxemia through exposure to immobilized polymyxin B without the systemic toxicity. This method and others [181] are being actively explored in clinical trials.

Cruz *et al*. [171] systematically reviewed the polymyxin-B hemo-perfusion literature published to 2006 and found 28 publications. The results of these studies were heterogeneous. Many were small, non-randomized and unblinded studies. Also, there was possible duplicate publication amongst these studies. All but two of the 28 publications had come from groups in Japan although since 2006 several studies from European centres have been published.

Whilst the Japanese studies suggest that levels of endotoxemia were reduced by polymyxin-B hemo-perfusion and that there was a reduction in mortality of nearly 50% [172], the experience among several small European studies has been inconsistent. These European studies, which all had fewer than 70 patients, have tested for either reductions in endotoxemia or mortality using polymyxin-B based or other hemo-perfusion interventions [173,174,175,176,177]. These studies found either no mortality benefit with a reduction in endotoxemia [173,174], or a reduction in mortality with an unknown effect on endotoxemia [175], or neither finding [176,177].

The most promising result of all studies came from the EUPHAS study (Early Use of Polymyxin Hemoperfusion in Abdominal Septic shock) which studied post-surgical patients with severe sepsis or septic shock secondary to intra-abdominal infection. This study was conducted in 10 Italian ICU’s over a 3 year period (2004–2007) and was terminated after an interim analysis revealed that a statistically significant and seemingly impressive reduction in clinical severity indices and mortality had become apparent between the groups after the enrolment of 64 patients. The reduction in 28 day mortality achieved in this study was as follows; 11 of 34 (33%) patients with polymyxin-B hemo-perfusion *versus* 16 of 30 (53%) patients with conventional therapy [175].

However, the interpretation of the EUPHAS study is unclear for several reasons [178,179,180]. One is that even with 64 patients, it is a small study. Also, the number of bacteremias found in the two groups was unequal; 16 among the patients receiving polymyxin-B hemo-perfusion *versus* only 3 among the patients receiving conventional therapy, a highly significant and unexplained imbalance. Hence whether the efficacy of the polymyxin is dependent on the clearance of endotoxemia or of bacteremia remains unclear. A third criticism is that survival had been prolonged by a few days only. In this regard the study results were statistically significant only according to an analysis based on the relative survival time (adjusted hazard ratio 0.36; 95% CI: 0.16–0.8) but not by an analysis of absolute survival (Fisher’s exact test; *p* = 0.13) A final criticism of the EUPHAS study is that endotoxemia levels were not measured. These study deficiencies are to be addressed in two follow up multi-center studies, the EUPHRATES (Evaluating Use of Polymyxin Hemo-perfusion in an RCT of Adults treated for Endotoxemia and Septic shock) which is to be undertaken in North American ICU’s together with the EUPHAS2 which is to be undertaken in Italian centers.

Another European study of endotoxemia adsorption using hemo-perfusion randomized 143 septic patients with suspected Gram-negative sepsis to receive either standard therapy alone *versus* standard therapy plus an extracorporeal endotoxin absorber. In this study [181], the endotoxin absorber was immobilised human serum albumin (iHSA), not polymxin. There was a non-significant trend toward lower endotoxemia levels with the extracorporeal endotoxin absorber treatment but no difference was found in the primary end point overall (APACHEII score) or survival.

Hence despite proven *in vitro* efficacy, the clinical efficacy of polymyxin remains unclear in clinical studies. Moreover, whether any clinical efficacy is contingent on reduction in detectable levels of endotoxemia *versus* on anti-bacterial effects for example also remains unclear.

## 6. Conclusions

Endotoxin is an attractive target against which to develop adjuvant therapies such as antibodies to aid in the management of sepsis. However, there are multiple challenges toward the development of these therapies. The first is the design of suitable experimental models with which to test the effects of anti-endotoxin antibodies. The extreme sensitivity of humans to endotoxin *versus* laboratory animals and the relative anti-bacterial *versus* anti-endotoxin effects of candidate antibodies are major considerations here. More broadly, the exact role of lipid-A and the importance of endotoxemia in the mediation of sepsis both remain to be clarified. The disconnect has increased with the recent progress in understandings of the complexities of the lipid-A structure function relationship on the one hand and the heterogeneous relationship between endotoxemia detection and the outcome of sepsis on the other. For these reasons, endotoxemia, as with GN bacteremia, should no longer be considered as a single entity. Moreover, the pathophysiological relevance of endotoxemia is dependent on the presence and type of concomitant GN bacteremia and possibly also on the underlying patient risk. However, an overarching challenge toward the further development of anti-endotoxin antibodies, as with other anti-endotoxin therapeutics, is the need to acknowledge the substantial and increasing disconnect between the pre-clinical *versus* clinical experience.
